# Computer-aided diagnosis through medical image retrieval in radiology

**DOI:** 10.1038/s41598-022-25027-2

**Published:** 2022-12-01

**Authors:** Wilson Silva, Tiago Gonçalves, Kirsi Härmä, Erich Schröder, Verena Carola Obmann, María Cecilia Barroso, Alexander Poellinger, Mauricio Reyes, Jaime S. Cardoso

**Affiliations:** 1 INESC TEC, Porto, Portugal; 2 Faculty of Engineering, University of Porto, Porto, Portugal; 3 Department of Diagnostic, Interventional and Pediatric Radiology, Inselspital, Bern University Hospital, University of Bern, Bern, Switzerland; 4 ARTORG Center for Biomedical Engineering Research, University of Bern, Bern, Switzerland

**Keywords:** Biomedical engineering, Computer science, Diagnosis

## Abstract

Currently, radiologists face an excessive workload, which leads to high levels of fatigue, and consequently, to undesired diagnosis mistakes. Decision support systems can be used to prioritize and help radiologists making quicker decisions. In this sense, medical content-based image retrieval systems can be of extreme utility by providing well-curated similar examples. Nonetheless, most medical content-based image retrieval systems work by finding the most similar image, which is not equivalent to finding the most similar image in terms of disease and its severity. Here, we propose an interpretability-driven and an attention-driven medical image retrieval system. We conducted experiments in a large and publicly available dataset of chest radiographs with structured labels derived from free-text radiology reports (MIMIC-CXR-JPG). We evaluated the methods on two common conditions: pleural effusion and (potential) pneumonia. As ground-truth to perform the evaluation, query/test and catalogue images were classified and ordered by an experienced board-certified radiologist. For a profound and complete evaluation, additional radiologists also provided their rankings, which allowed us to infer inter-rater variability, and yield qualitative performance levels. Based on our ground-truth ranking, we also quantitatively evaluated the proposed approaches by computing the normalized Discounted Cumulative Gain (nDCG). We found that the Interpretability-guided approach outperforms the other state-of-the-art approaches and shows the best agreement with the most experienced radiologist. Furthermore, its performance lies within the observed inter-rater variability.

## Introduction

The increasing use of advanced cross-sectional imaging and the evolution of the information technology infrastructure to meet the demands of higher imaging volumes (i.e., improved computational power, storage capacity, and workflow efficiency in the picture archiving and communication system (PACS) environment), contributed to a substantial increase of the amount of images generated per examination^1^. Consequently, this has increased the workload of radiologists, which must now interpret more examination images in less time, thus creating the possibility for increased detection errors as a result of increased fatigue and stress, lowering the quality of the healthcare delivered by the radiologists to the patients^2,3^. Moreover, as the ratio of diagnostic demand to the number of radiologists increases, the diminished effective available time per diagnostic becomes a critical issue^4^. According to the current paradigm, in case of doubt for a suspected condition, radiologists often turn to public or internal image databases where similar disease-matching images of the diseases the radiologist has narrowed down can be searched and compared against (e.g., *Radiopaedia*). After reviewing all possible differential diagnoses (those originally considered and those that came up during the search), the radiologist weighs these diagnoses and usually gives 2–4 of them as possible diagnoses. In this process, the radiologist ranks the images and creates an ordered set of images in his/her head. This task is time-consuming and often ineffective since it requires several iterations until a proper matching image supporting the final diagnosis is found. Moreover, these databases are limited in the variability of cases presented to the users, which is exacerbated in conditions of low prevalence. Hence, it is extremely relevant to develop disease-targeted content-based image retrieval (CBIR) systems that automatically present disease-matching similar images to the one being analysed. A CBIR system usually focuses on two different tasks: feature representation, which consists of finding a low-dimensional representation of the image that is suitable for characterising it well enough; and, feature indexing and search, which focus on the efficiency of the retrieval process^5^. Our work focuses on the first step, i.e., on finding the most appropriate feature representation for the task at hand.

**Figure 1:**
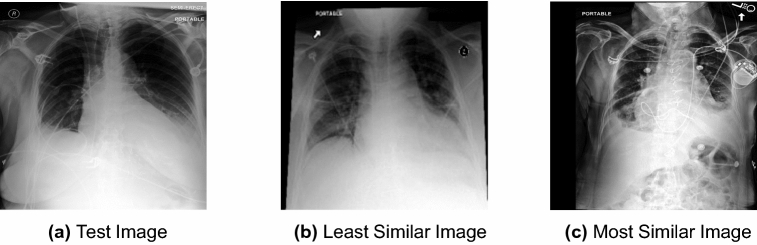
Pleural Effusion test image and the least and most similar images of the catalogue according to our board-certified radiologist (in terms of disease and disease severity). The overall most similar image would be (**b**). However, such matching is not of radiological interest.

Finding the most appropriate feature representation is an arduous task since the clinical analysis is typically constricted to a small region of the image, discarding most of the available information. As such, finding the overall most similar image (i.e., including all pixels in the image) is not the objective, instead, we are interested in finding the most similar image in terms of disease and disease severity. As illustrated in Fig. 1, those can be quite far apart, as Fig. 1b is, overall, more similar to Fig. 1a than Fig. 1c, while in terms of disease and disease severity, it is the opposite, with Fig. 1b being the least similar image and Fig. 1c the most similar (from a catalogue of 10 images).

Given that the disease features are located in a small region of the entire image, the medical CBIR system should also be paying attention to that specific region, ignoring the remaining information. However, most CBIR systems perform their analysis taking the entire image into account, particularly the more traditional methods. Deep learning approaches have a better focus on the disease-related characteristics as they learn the appropriate feature representations to solve the classification task of interest. Thus, they represent an improvement in terms of focus when compared to the more traditional approaches. Nonetheless, we hypothesise that this can be further improved by increasing even more the focus of the network in the regions that matter to the decision, and explore two different techniques: one driven by interpretability, and another based on attention mechanisms.

This paper builds upon our work proposed in Silva et al.^4^. In this study we extend our previous work by (1) adding experiments with a second new dataset; (2) a second medical condition (pneumonia), and (3) a comparison to a recently proposed network, employing implicit attention mechanisms. Furthermore, we improved our comparisons to expert radiologists by adding two more board-certified radiologists to each study in order to assess the evaluated methods with respect to the inter-rater variability of these tasks.

The remainder of this paper is organised as follows: section “Background” introduces the concepts and state-of-the-art of the topics related to this study, namely, Medical Image Retrieval, Explainable Artificial Intelligence, and Attention Mechanisms; section “Materials and methods” describes the dataset used, the baselines, our methods, and the evaluation framework; section “Results and discussion” presents the quantitative and qualitative results obtained for the two conditions (pleural effusion, and pneumonia), and also a discussion of those results; section “Conclusions” sums up the conclusions drawn from this work and suggests new directions for future work in this research area.

## Background

### Medical image retrieval

The importance of having a good medical image retrieval system to help clinicians make a diagnosis was clearly pointed out in the previous section. Here, we will focus on presenting the most relevant CBIR works available in the literature. The main difficulties in the development of CBIR systems are related to the development of algorithms that generate useful semantic representations of medical images in order to effectively retrieve the most similar examples^6^, and on the integration of these algorithms in end-user applications^7,8^. We will focus on the first difficulty. In that regard, several works were presented in the literature to find the most suitable representation to perform the retrieval: Tizhoosh^9^ explored the use of bar code annotations as an auxiliary method for feature-based image retrieval; Srinivas et al.^10^ implemented a clustering method that uses dictionary learning to group large medical databases and relies on different similarity measures (e.g., Euclidean) to perform image retrieval; Hofmanninger and Langs^11^ proposed the re-mapping of visual features extracted from medical imaging data based on weak labels to obtain descriptions of local image content capturing clinically relevant information; Seetharaman and Sathiamoorthy^12^ presented a unified learning framework for heterogeneous medical image retrieval based on a full range auto-regressive model with a Bayesian approach to extract meaningful image features; Ma et al.^13^ created a method that consists of a weighted graph whose nodes represent the images and edges measure their pairwise similarities; Nowaková et al.^14^ presented a novel method for fuzzy medical image retrieval using vector quantisation with fuzzy signatures in conjunction with fuzzy S-trees; Qayyum et al.^15^, Ayyachamy et al.^16^ and Owais et al.^17^ trained CNNs on multimodal and multi-class data sets, and used the learned features and the classification results to retrieve medical images; Cai et al.^18^ used a Siamese Network in the learning process, with the CNN of each branch being used to extract features, followed by the application of a binary hash-mapping to reduce the dimensions of the feature vectors; Minarno et al.^19^ used a CNN-based auto-encoder method in the feature extraction process to improve the results of the retrieval process; Mbilinyi et al.^20^ used a deep metric learning approach and the triplet loss to learn a model that receives an image and a text description highlighting specific diagnoses the retrieved images should have. In summary, feature representation is performed in one of the following ways: statistical measures, hand-crafted features, learned features, or a combination of the previously mentioned strategies. However, to the best of our knowledge, none of the previously proposed approaches explicitly focuses the training process on the disease-related characteristics without requiring additional labels. In this work, we aim to utilize AI interpretability methods to guide the retrieval process, with focus on the disease and without necessitating any additional related label information.

### Explainable artificial intelligence

In the last years, deep learning algorithms have been highly successful in medical image applications, in some cases even challenging human performance^21^. Nonetheless, both clinical and technical communities acknowledge that there are still several open challenges that need to be addressed. Particularly of interest for this study are the works that try to overcome the transparency and trust issues. As pointed out, most of these complex and successful models currently used to solve medical imaging problems work as black boxes (i.e., their internal logic is hidden to the user), without being able to explain their predictions in a human-understandable way^22^. Despite being a research field under development, there are already many approaches to obtain interpretability, or in a broader sense, to produce explanations for the decisions that models make. Interpretability research can be easily understood by looking at the three-stage categorization (pre-model, in-model and post-model) proposed by Kim and Doshi-Velez^23^. Pre-model methods focus on understanding the data distribution before building the model through exploratory data analysis^24–27^. In-model methods seek to integrate interpretability inside the model, either by relying on models based on rules^28,29^, based on cases^30–32^, through the use of regularization techniques (e.g., sparsity, monotonicity) during training^33,34^, by guiding the neural network into learning relevant concepts^35,36^, or by seeking to integrate causal knowledge into the network^37,38^. Finally, post-model methods are related to a posterior analysis of the model predictions, either producing saliency maps through gradient information^39–41^, deconvolution^42,43^, optimization^44^, decomposition^45,46^, or through a connection with high-level semantic concepts^35,47,48^. In this work, we will focus on post-model interpretability strategies, as we are interested in finding the most relevant regions for the medical decisions (explicit attention) without limiting in any way the learning process nor requiring any additional label. This can be done by identifying the areas of the image that mostly contribute to the final decision. To find these relevant regions, we used *Deep Taylor*^46^, which is a relevance propagation approach (similar to Layer-wise Relevance Propagation (LRP)^45^), that uses deep Taylor decomposition to efficiently assess the importance of single pixels in image classification problems. The choice of this interpretability method in specific was mainly driven by its recognized quality, but also because it was the method that produced the saliency maps more in-line with what our board-certified radiologist considered as relevant medical information.

### Attention mechanisms

A different alternative to the use of post-hoc interpretability methods to focus the network into the disease-related characteristics is the use of implicit attention mechanisms. This application of attention mechanisms in deep learning algorithms was inspired by the field of psychology, according to which humans tend to selectively concentrate on a part of the information^49^. For instance, the human visual system tends to selectively focus on specific parts of an image while ignoring others^50^. The use of attention was initially proposed in Bahdanau *et al.*^51^, for the task of neural machine translation. In this work, the authors use an encoder-decoder architecture presenting two challenges: (1) the decoder needs to compress all the input information into a single fixed-length vector and pass it to the decoder; (2) ensuring model alignment between input and output sequences was not possible. Hence, it was necessary to develop an attention mechanism that could support the decoder in focusing on the relevant parts of the inputs^52^. Naturally, during the training phase, an extra task is added: the learning of the attention weights. Nevertheless, this approach showed improved results against the state-of-the-art and paved the way for the creation of novel attention-based methodologies. Attention models can be classified into different categories according to their input sequences, output sequences, candidate states (hidden states of the encoder) and query states (hidden states of the decoder)^52^. Of relevance for this work are self-attention and multi-level attention, with self-attention being when the query and candidate states belong to the same input sequence, and multi-level attention when we apply the attention mechanism on multiple levels of abstraction of the input sequence. Additional details on the attention mechanisms used in this work will be presented later when discussing their application in content-based image retrieval.

## Materials and methods

### Data

For the experiments, we used the MIMIC Chest X-ray JPG, which is a large and publicly available database. It consists of chest radiographs already converted to JPG format, and their respective labels, which were derived from free-text radiology reports^53^. In this database, there are 377,110 JPG format chest radiographs with associated structured labels. Institutional approval was granted for the use of the patient datasets in research studies for diagnostic and therapeutic purposes. Approval was granted on the grounds of existing datasets. All methods were carried out in accordance with relevant guidelines and regulations. From all the available labels, we focused our attention on the following conditions: Pleural Effusion, and (Potential) Pneumonia. Even though the provided label is pneumonia, our main board-certified radiologist considers a Chest X-ray does not allow a conclusive diagnosis of pneumonia, thus, we perform our analysis referring to the cases as having potential pneumonia. To train and evaluate our models, the original splits selected by the data providers^53^ were used, i.e., we considered the training fold for training, the validation fold to select the final model, and the test set to evaluate the performance. From these splits, we only considered frontal view images, either acquired in an AP (Anterior-posterior) or PA (Posterior-anterior) setting. For completeness, one of the analysis was done with AP view images (pleural effusion) and the other with PA view images (potential pneumonia). For each condition there are four possible annotations: 1, the label was positively mentioned in the associated study; 0, the label was negatively mentioned in the associated study; − 1, the label was either mentioned with uncertainty or with ambiguous language in the report; and missing, no mention of the label was made in the report. Regarding our experiments, we only considered images associated with the labels 1 and 0, thus, not introducing uncertainty in the training of the models. Given that selection, we ended up with 61203 training images, 534 validation images, and 1072 test images for pleural effusion and 18226 training images, 133 validation images, and 258 test images for pneumonia.

**Figure 2:**
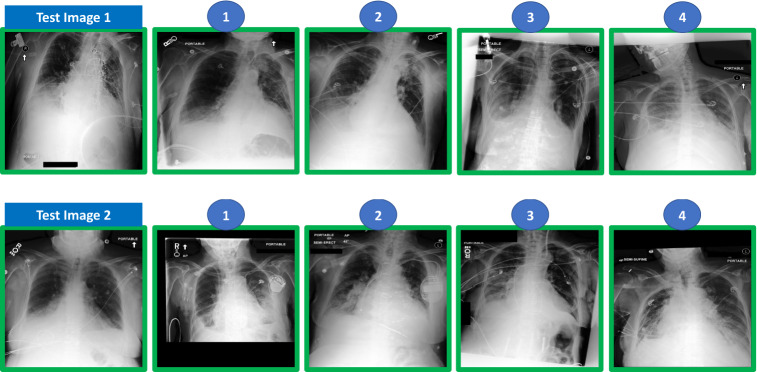
Example of test cases and ranking annotation for pleural effusion condition (the top 4 is shown) performed by the radiologist. The numbers on top of the image represent the ranking position of the image in the catalogue (when compared to the query/test image). The green box means pleural effusion case (according to the dataset label).

**Figure 3:**
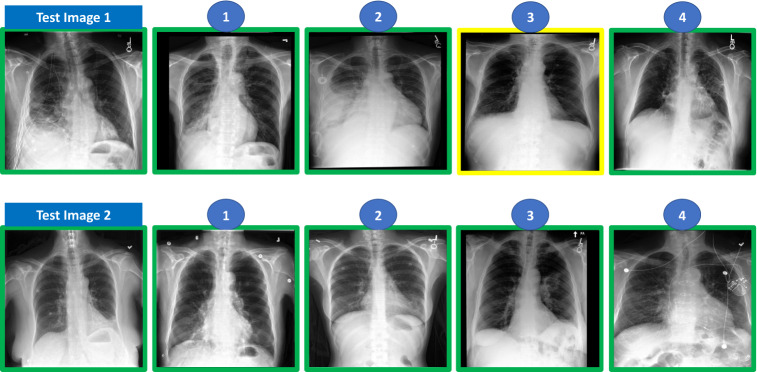
Example of test cases and ranking annotation for potential pneumonia condition (the top 4 are shown) performed by the radiologist. The numbers on top of the image represent the ranking position of the image in the catalogue (when compared to the query/test image). The green box means pneumonia case (according to the dataset label), yellow box means radiologist considers case as potential pneumonia and dataset label is no pneumonia.

To evaluate the performance of the different types of methods in the retrieval task, we split the test data into query and catalogue images. For pleural effusion, we considered ten query images, each associated with ten catalogue images. For pneumonia, the test set was considerably smaller, and we only considered five query images, each associated with also ten catalogue images. For both conditions, query and catalogue images were randomly chosen. All images considered for the evaluation were labelled by our main board-certified radiologist. Besides labelling all images, our main radiologist also ranked the associated catalogue images in relation to each of the test images, considering the similarity in terms of disease severity (Fig. 2 shows an example of the ranking annotations provided for pleural effusion and Fig. 3 shows an example of the ranking annotations provided for pneumonia). In addition to our main board-certified radiologist, four other experienced radiologists also analysed and ranked the cases in order to assess the inter-rater variability in such a task.

### Methods

#### Structural similarity index (SSIM)

The first method to be considered for evaluation in the retrieval task is the classic statistically-based structural similarity index (SSIM)^54^. As in Silva et al. ^4^, the SSIM was computed directly between test and catalogue images, using its default values. Since higher SSIM values represent higher similarity, the top retrieved image is the one with the highest similarity index.

#### Convolutional neural network (CNN)

The second method to be considered is already a deep learning based method, where the relevant features are automatically identified^5,11,55,56^. As in Silva et al.^4^, we use the DenseNet-121^57^ as our CNN architecture. However, in this work, we do not initialize its weights with the ImageNet pre-training, but instead with the pleural effusion CheXpert CNN model from Silva et al.^4^ for the pleural effusion condition, and with the pleural effusion model from this work for the pneumonia condition, as pre-training using data more similar to the final domain is more effective than using ImageNet pre-training^58,59^. Similarity between images is computed based on the Euclidean distance in the feature space of the previous to the last layer of the model. Since shorter distances represent higher similarity, the top retrieved image is the one with the shortest distance to the test image. The distance between two images is formalized in Eq. (1), where $I_{t}$ represents the test image *t*, $I_{c}$ represents the catalogue image *c*, $\theta _{\text {CNN}}$ represents the CNN model parameters, and *F* represents the function that translates the original image into a latent representation constituted by the features in the previous to last layer of the network (i.e., in a vector of dimension 1024).

1 $$\begin{aligned} {d_{CNN}}({I_{t}, I_{c}}) = || F(\theta _{\text {CNN}}, {I_{t}}) - F(\theta _{\text {CNN}}, {I_{c}}) ||_{2} \end{aligned}$$

#### Interpretability-guided network (IG)

**Figure 4:**
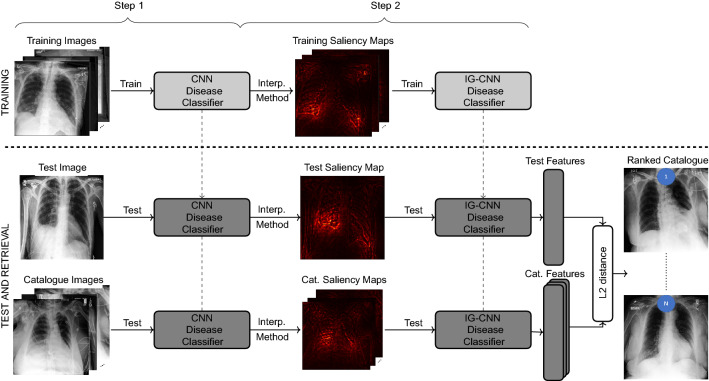
Overview of the proposed Interpretability-guided approach. Blocks in light gray () mean neural networks are being trained (i.e., weights are being updated), whereas blocks in dark gray () represent trained neural networks (i.e., weights are fixed). In the saliency maps, brighter colors mean higher relevance. Blue circles indicate ranking positions. CNN represents the deep model used as baseline. IG-CNN represents the CNN model architecture being trained with saliency maps. The L2 distance is computed between the test image’s latent features and each catalogue image’s latent features.

The third method being considered is the method proposed in Silva et al.^4^. It uses the exact same architecture as the CNN model, but has as input the saliency maps, instead of the original images (Fig. 4) in order to focus the network into the disease-related characteristics. Those saliency maps are computed using the Deep Taylor interpretability method^45^, and are based on the previously presented CNN model. This time, the deep learning network was initialized with the CheXpert IG model from Silva et al.^4^ for the pleural effusion condition, and with the IG pleural effusion model from this work for the pneumonia condition. As with the CNN approach, the similarity is computed based on the previous to last layer of the model. The distance between two images is formalized in Eq. (2), where $I_{t}$ represents the test image *t*, $I_{c}$ the catalogue image *c*, $\theta _{\text {CNN}}$ the CNN model parameters, $\theta _{\text {IG}}$ the IG parameters, *S* the function that generates the saliency maps, and *F* the function that translates the original image into a latent representation constituted by the features in the previous to last layer of the network.

2 $$\begin{aligned} {d_{IG}}({I_{t}, I_{c}}) = || F(\theta _{{IG}},S(\theta _{{CNN}}, {I_{t}})) - F(\theta _{{IG}},S(\theta _{{CNN}}, {I_{c}})) ||_{2} \end{aligned}$$

#### Attention network (ATT)

Here, we add a fourth method to the comparison, one that is driven by attention mechanisms. Recently, a CNN with a multi-level dual-attention mechanism (MLDAM) has been proposed for macular optical coherence tomography classification^60^. The main novelty of this work in the context of medical image classification is the joint application of a *self-attention* and a *multi-level attention* mechanisms that allow the network to learn relevant features in coarser as well as finer sub-spaces. In their article^60^, the authors state that this technique enables the network to utilise the information of coarser features preventing loss of any useful information, thus enabling the network to yield more focused features and better convergence. Regarding the impact of the application of attention mechanisms in the interpretability of deep learning algorithms, Chen and Ross^61^ proposed the joint use of a position attention module (PAM) and a channel attention module (CAM) to refine the pixel values at spatial and channel levels. These refined features are then fused through an element-wise sum. The authors performed an analysis of the saliency maps produced by the gradient-weighted class activation mapping (Grad-CAM)^41^ and concluded that the use of attention modules had enabled the network to shift the focus on to the annular iris region.

In this work, we aimed to assure diversity in the levels and scales of the features extracted from the DenseNet-121^57^. Following the notation in Mishra et al.^60^, let $I_{A}$, $I_{B}$ and $I_{C}$ be the multi-level features extracted from the backbone. We extracted features from different dense-blocks resulting in a $I_{A}$ with shape $\left[ 512, 28, 28 \right]$, a $I_{B}$ with shape $\left[ 1024, 14, 14 \right]$ and a $I_{C}$ with shape $\left[ 1024, 7, 7 \right]$.

In line with the previous deep methods, the similarity is computed by measuring the Euclidean distance in the previous to last layer. The distance between two images is formalised in Eq. (3), where $I_{t}$ represents the test image *t*, $I_{c}$ the catalogue image *c*, $\theta _{\text {ATT}}$ the Attention model parameters, and *A* represents the function that that translates the original image into a latent representation constituted by the features in the previous to last layer of the network.

3 $$\begin{aligned} {d_{ATT}}({I_{t}, I_{c}}) = || A(\theta _{{ATT}},{I_{t}}) - A(\theta _{{ATT}},{I_{c}}) ||_{2} \end{aligned}$$

### Deep learning networks training

All deep learning methods (i.e., CNN, IG, and ATT) were trained to solve binary classification tasks (e.g., pleural effusion vs. non-pleural effusion). Thus, we use the binary cross-entropy as our loss function (Eq. 4, where *y* is the binary indicator, *ln* the natural logarithm, *p* the predicted probability, and $\theta$ the model parameters).

4 $$\begin{aligned} {\mathscr L}(\theta ) = - (y \ln (p(\theta )) + (1-y) \ln (1-p(\theta ))) \end{aligned}$$

For the pleural effusion condition, the deep learning models were trained for 10 epochs, with a batch size of 32, and using the *Adadelta* optimiser^62^. Since the data for the pleural effusion condition is highly imbalanced, the misclassifications were weighted with the inverse of the frequency of the respective class to promote a similar focus of the network in both classes^63^.

Regarding the pneumonia condition, the deep learning models were trained for 15 epochs, with a batch size of 32, and using the *Adam* optimiser^64^ with a learning rate $l_{r}$ = 1$\times$ $10^{-4}$. The *Adam* optimiser was chosen over the *Adadelta* due to converging issues during the training of the CNN model, and was kept for the training of the other deep models (IG and ATT) for consistency.

For both conditions, small rotations and translations were used as data augmentation. Hyperparameter values were empirically optimised for the CNN models and replicated for all the others. Final models were selected based on the F1 score (Eq. 5) in the validation set.

5 $$\begin{aligned} \text {F1} = 2 \times \dfrac{\text {precision} \times \text {recall}}{\text {precision} + \text {recall}} \end{aligned}$$

We note that the training process was agnostic to the ranking task at hand. No information of ranking was provided at any point, neither in the loss function nor in the selection of the best performing model in validation.

The methods were implemented using Keras^65^ with TensorFlow backend in a workstation equipped with an NVIDIA Tesla V100 (32 GB) GPU. For the generation of the saliency maps, we used the *iNNvestigate* toolbox^66^ implementation of the Deep Taylor method, as in Silva et al.^4^.

### Evaluation and comparison

The quality of the retrieval is evaluated by computing the normalised Discounted Cumulative Gain (nDCG)—Eq. (6), which is the normalised version of the Discounted Cumulative Gain (DCG)—Eq. (7), being it a common metric in learning to rank tasks^67^. The normalisation is done over the maximum possible value of the DCG metric (in our work, the maximum possible value is obtained when the ranking of the method is exactly the same as our ground-truth). The subscript *p* represents the number of retrieved images we are considering for the evaluation (e.g., when we perform the evaluation over the entire set of retrieved images, *p* = 10). In Eq. (7), $rel_{i}$ represents the relevance value assigned to the ranking position *i*, with the least similar image having relevance of 1 and the most similar image having relevance of 5.5 (i.e., the relevance of two contiguous positions differs by 0.5). Thus, the first positions of the catalogue ranking have more importance than the last ones, with the importance being gradually reduced as we go from the first to last ranked image.

6 $$\begin{aligned} \text {nDCG}_{p}= & {} \dfrac{DCG_{p}}{IDCG_{p}} \end{aligned}$$

7 $$\begin{aligned} \text {DCG}_{p}= & {} \sum _{i=1}^{p} \dfrac{2^{rel_{i}} - 1}{\log _2(i+1)} \end{aligned}$$

In order to contextualize the retrieval results of our machine learning methods, we also asked our partner board-certified radiologists to provide their similarity rankings for the pleural effusion and pneumonia conditions. Thus, we are able to check the inter-rater variability in ranking tasks, also helping us to have a more complete evaluation of our methods’ quality.

## Results and discussion

### Pleural effusion

Our first experiments were conducted for the pleural effusion condition. All images used here were frontal X-ray images acquired in an AP view fashion. Thus, experiments were performed with 61203 training images, 534 validation images, and 1072 test images. The training images were used to find the optimal set of parameters, the validation images to select the final classification model, and the test images for the assessment. To evaluate the ranking quality, ten different query images and ten catalogues of ten images each were randomly created, splitting the test data into query and catalogue images by using ten different random seeds (keeping the proportion of the classes). Afterwards, our main board-certified radiologist provided us with a ranking of those ten images in relation to the respective query image, serving as our ground-truth ranking. Moreover, we also asked two other board-certified radiologists to provide their rankings in order to compare inter-rater variability with our models’ performance.

**Figure 5:**
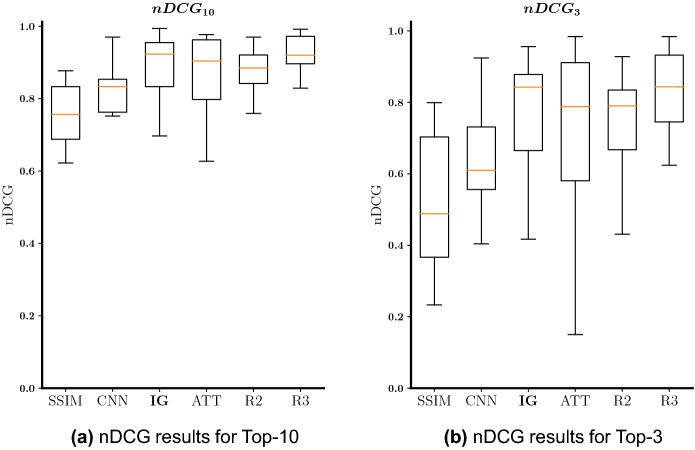
Box-and-whisker plots regarding the nDCG results for the pleural effusion Top-10 (**a**) and Top-3 (**b**) retrieved images. SSIM is the statistically-based baseline, CNN is the CNN-based baseline, IG is the proposed interpretability-guided approach, ATT is the attention method, R2 is the ranking provided by the second board-certified radiologist, and R3 is the ranking provided by the third board-certified radiologist.

In Fig. 5a, we present the nDCG results obtained with the statistical and machine learning models (i.e., SSIM, CNN, IG, and ATT) and also the results obtained by considering the rankings provided by two other radiologists (R2, and R3) for the Top-10 retrieved images. By observing the box-and-whisker plot, we conclude that the proposed interpretability-guided approach (IG) and the attention-based method (ATT) are the ones that lead to the best nDCG results for the Top-10 retrieved images, with the interpretability-guided approach outperforming the attention-driven method. Those results are in line with those from the other radiologists, demonstrating the high-quality of both methods. Furthermore, the CNN approach leads to better results than the SSIM method, as it was expected. The same can be observed in Fig. 5b, where the nDCG results for the Top-3 retrieved images are presented (in clinical practice having the three most similar images is typically enough to help the radiologist make the diagnosis). In this scenario, nDCG values are worse than in the previous experiment due to only considering the Top-3 retrieved images, highly penalizing a “failure” in one or more of these images. This also contributed to an increase in the variability of the results obtained, particularly in the case of the ATT method. Nonetheless, IG and ATT approaches remained the best methods and are still in line with the performance of the two radiologists.

**Figure 6:**
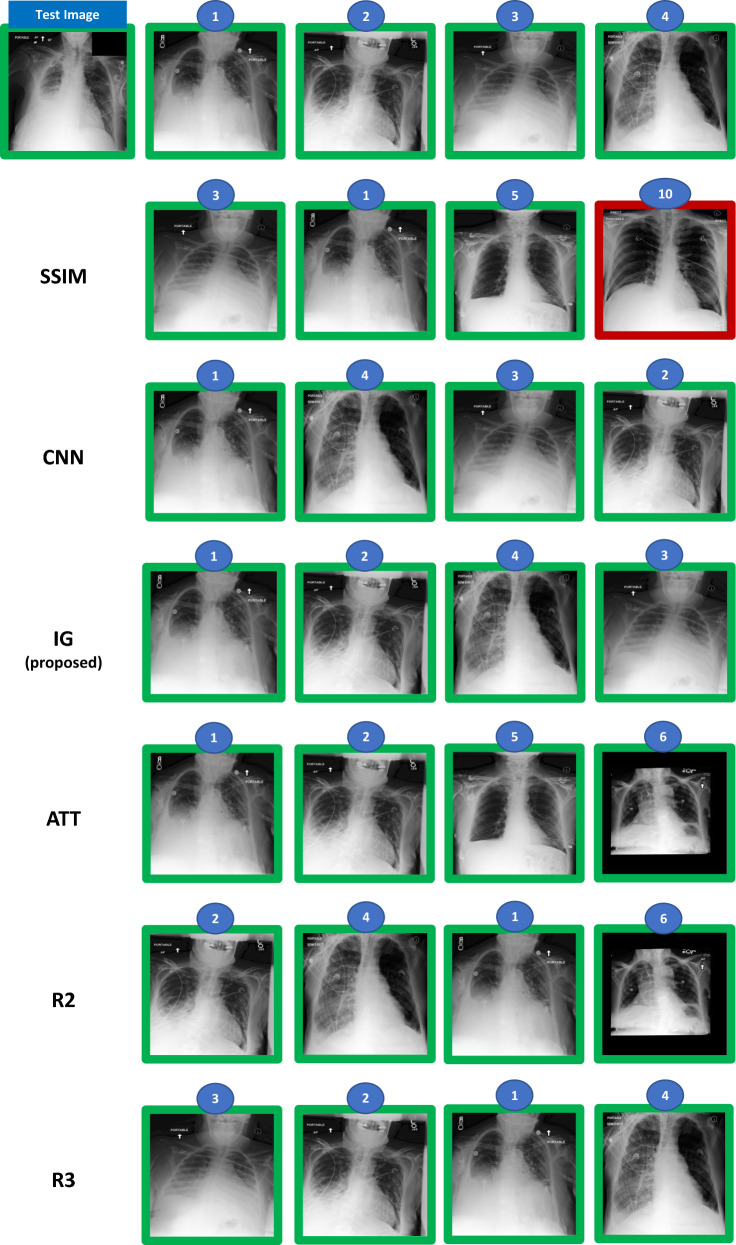
Example of test case and the Top-4 retrieved images given by each of the radiologists (R1 = ground-truth, R2, and R3) and each of the machine learning methods. In this split, both the CNN, IG, and ATT obtained nDCGs (Top-10) > 0.9. The green box means pleural effusion case and the red box means no pleural effusion (according to the dataset label).

In Fig. 6, we show the Top-4 retrieved results obtained by each of the methods, and provided by the radiologists in comparison with the ground-truth defined by our main radiologist for one split, corresponding to a specific test case and catalogue. In this split, all machine learning methods (i.e., CNN, IG, and ATT) attained extremely high nDCG results. Both CNN and IG retrieved the same Top-4 images, with the only difference being the ranking of these four images, with IG’s ranking being closer to the one provided by our main radiologist than CNN’s ranking. Even though the ATT’s Top-4 retrieved images differ from the ones selected by our main radiologist, one of those images was also selected by one of the other radiologists (i.e., R2). SSIM was the worst method, selecting the least similar image (a non-pleural effusion case) for the Top-4 retrieved images.

In terms of classification performance in the entire test set (the 1072 test images), the interpretability-driven model was the one leading to the best results (F1-score = 0.862), followed by the attention-based model (F1-score = 0.809), and the standard CNN model (F1-score=0.790).

### (Potential) Pneumonia

**Figure 7:**
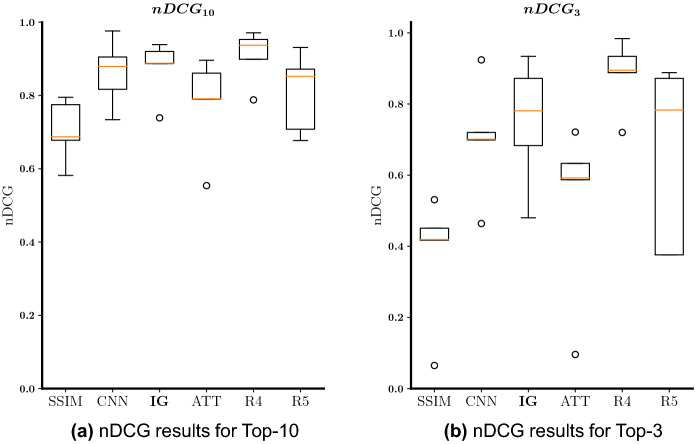
Box-and-whisker plots regarding the nDCG results for (potential) pneumonia Top-10 (**a**) and Top-3 (**b**) retrieved images. SSIM is the statistically-based baseline, CNN is the CNN-based baseline, IG is the proposed interpretability-guided approach, ATT is the attention method, R4 is the ranking provided by the fourth board-certified radiologist, and R5 is the ranking provided by the fifth board-certified radiologist.

The following experiments were conducted for the pneumonia condition. All images used here were frontal X-ray images acquired in a PA view fashion. Thus, for the experiments we considered 18226 training images, 133 validation images, and 258 test images. For the ranking evaluation, five different query images and five catalogues of ten images each were created, splitting the data into query and catalogue images by using five different seeds (keeping the proportion of the classes). Afterwards, the catalogue’s ten images were ranked in terms of their potential as pneumonia cases to the respective query image. Even though our dataset annotations for training and validation were pneumonia annotations, our main radiologist considers a Chest X-ray as only indicative of potential pneumonia, and not of a definitive diagnosis. Thus, catalogue images were ranked having in mind their potential as pneumonia cases. In Fig. 7a, we present the nDCG results obtained with the statistical and machine learning models and also the results obtained by considering the rankings provided by two other radiologists (R4, and R5). By observing the box-and-whisker plot, we infer that the proposed interpretability-guided approach (IG) is the method with the best retrieval ranking performance. On the contrary, for this condition, the attention-based method (ATT) had a poor ranking performance, obtaining nDCG results that were worse than the ones obtained with our deep learning baseline method (CNN), and only surpassing the performance of the statistical baseline (SSIM). The relative performance of the four methods was the same when we measured the nDCG performance for the Top-3 retrieved images (as shown in Fig. 7b). IG’s results also fall within the inter-rater variability of the radiologists, which demonstrates the quality of the method.

**Figure 8:**
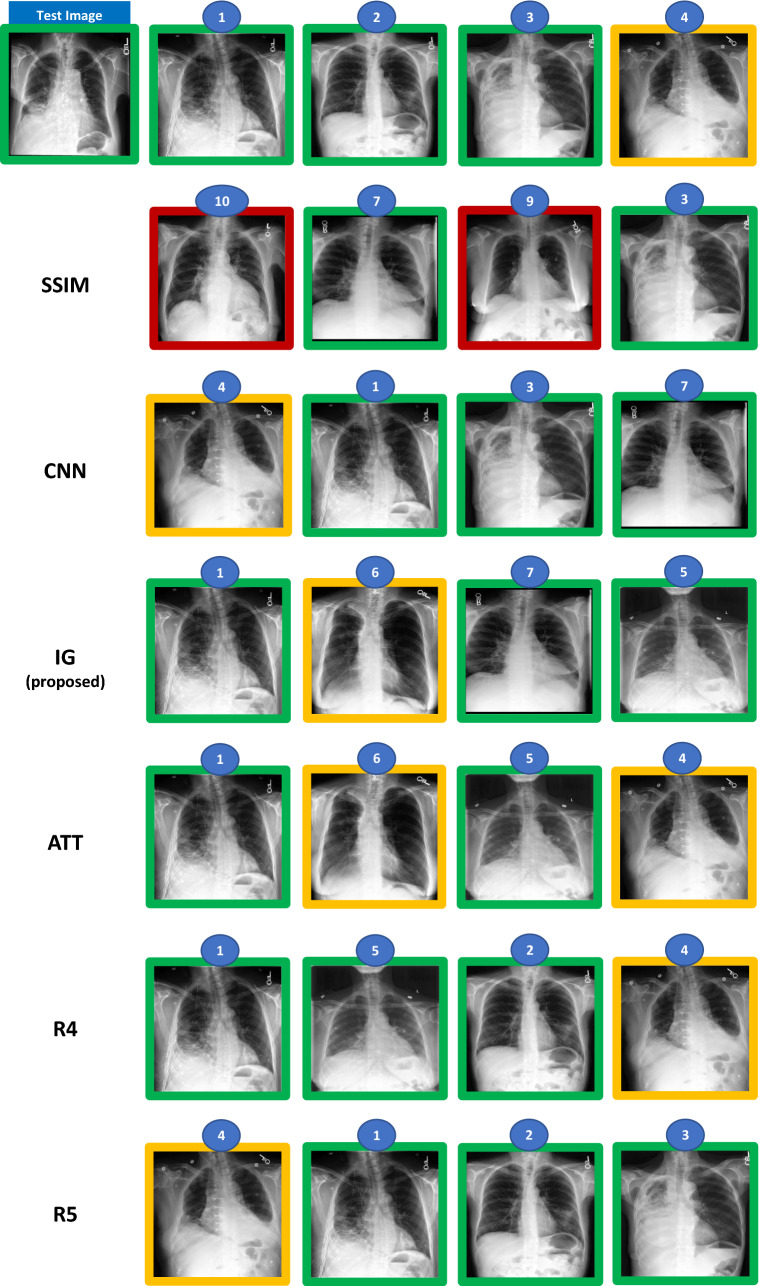
Example of test case and the Top-4 retrieved images given by each of the radiologists (R1 = ground-truth, R4, and R5) and each of the machine learning methods. In this split, both the CNN, IG, and ATT obtained nDCGs (Top-10) > 0.8. The green box means potential pneumonia case, red box means no potential pneumonia, and orange box means disagreement between R1 and label, with R1 considering the case as potential pneumonia.

When we compare the quantitative results obtained for the pneumonia condition with the ones obtained for the pleural effusion, we observe that they were considerably worse in general. That may be due to pneumonia being a more difficult to diagnose condition, and also to different interpretations of what a pneumonia Chest X-ray is (in several catalogue images, there was a disagreement between MIMIC-CXR label, and the diagnosis provided by our main board-certified radiologist). In Fig. 8, we present an example query case and the respective Top-4 retrieved images obtained by each of the methods, and provided by the radiologists in comparison with the ground-truth defined by our main radiologist. In this split, all deep learning models had a reasonably good ranking performance, with the interpretability-guided approach (IG), and the attention-based method (ATT) retrieving in the first position the most similar image in terms of pneumonia to the test image. As can be observed here, some images in this catalogue had different diagnoses given by MIMIC-CXR and by our main radiologist, namely the fourth and sixth ranking positions (images with orange boxes). Moreover, the direction of the disagreement was the same, with our main radiologist considering the cases as of potential pneumonia, and the MIMIC-CXR label being non-pneumonia. Nonetheless, even with this label disagreement, the performance obtained with our interpretability-guided approach (IG) was reasonably good, exceeding nDCG results of 0.88 for the Top-10 retrieved images in all but one split.

In terms of classification performance in the entire test set (the 258 test images), this time, the attention model was the one leading to the best results (F1-score = 0.655), followed by the interpretability-driven model (F1-score = 0.645), and the standard CNN model (F1-score = 0.620).

### Ablation study

**Figure 9:**
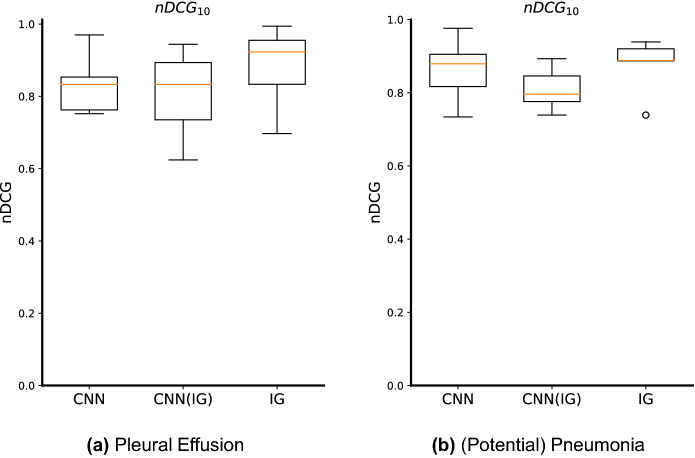
Box-and-whisker plots regarding the nDCG results for Top-10 retrieved images. CNN is the CNN baseline model, CNN(IG) is the CNN model having as inputs the Deep Taylor saliency maps, and IG is the proposed interpretability-based approach (i.e., it was trained (fine-tuned) with saliency maps, and has as inputs also saliency maps).

We also studied the relevance of training with the saliency maps, and not only using them to compute the features in the semantic space. In Fig. 9, we present the Top-10 nDCG results for both pleural effusion and potential pneumonia, considering the CNN baseline model and these two versions, i.e., using only the saliency maps as inputs to the CNN model—CNN(IG)—and our proposed method where we use the saliency maps both in the training and retrieval processes—IG. By observing Fig. 9, we conclude that the use of saliency maps in the training process helps to attain better results, which can be explained by an increase in the focus of the network on the relevant disease regions, and by learning this new saliency map distribution.

## Conclusions and future work

We have investigated the use of different content-based image retrieval methods in a Chest X-ray retrieval task, intending to study their potential to support a medical diagnosis. For radiologists, more important than having a decision support system providing prediction labels is to have a system that is able to present them with similar clinical cases, as it is usually the way they proceed when encountering a difficult diagnosis scenario. Moreover, radiologists feel more comfortable working with images than with textual descriptions, motivating the use of case-based reasoning or explainability.

For the medical image retrieval to be successful, the comparison between images has to take into account the particular nature of medical images, i.e., that the information of interest is commonly found in a specific region of the image, while the remaining information is irrelevant. Indeed, the structural similarity index method showed the poorest performance for both conditions, demonstrating that a general image comparison does not represent disease similarity. Driven by that notion, we proposed an interpretability-guided approach and investigated the use of attention mechanisms for the retrieval task. The proposed interpretability-guided medical image retrieval approach outperformed all the other studied methods for both considered conditions. Our approach has an explicit attention mechanism that is also more intelligible than the implicit attention mechanism of the attention-driven method, leading to a more interpretable solution. Moreover, it obtained a performance in line with other human experts (board-certified radiologists) for both conditions. In turn, the attention-based medical image retrieval method had an excellent performance for the pleural effusion condition (in line with both our proposed method and the other radiologists) but failed for the pneumonia condition.

It is important to emphasize that all methods were only trained to solve binary classification tasks, not using any ranking information. This means that the annotation effort required is significantly lower than if ranking information was also needed. Even though we did not use ranking information in the training process, our proposed approach correctly captured the ranking information, obtaining excellent nDCG results for both conditions. Test and catalogue images apart from being ranked were also labelled by our main board-certified radiologist. Particularly for the pneumonia condition, we observed some disagreements in the diagnosis, which may be indicative of the usage of different definitions, and may hinder the method’s performance. Nonetheless, even for the pneumonia condition, our proposed method obtained an excellent ranking performance.

This work aims to be the first step towards a deeper focus in medical image retrieval as a decision support system, helping radiologists make better and quicker decisions. However, further studies and investigations are required in order to translate these algorithms into the clinics. Considering the evaluation aspect, it is crucial to have more extensive studies, more datasets, other clinical problems, and more radiologists involved in the annotation and evaluation process. Regarding the technical side, there are several open problems or investigation opportunities, namely, the use of multimodal data, the introduction of causal knowledge^68^, privacy-preserving image retrieval^69^, and also exploring federated learning settings^70^. By the use of multimodal data, we mean the integration of the clinical reports in the learning process, also with the possibility of accompanying the top retrieved images with a generated clinical report to provide complementary information, which can be particularly interesting when the end-user is a general practitioner instead of a radiologist. In this work, we observed that by using post-hoc interpretability saliency maps, we were able to focus model attention into more clinically relevant regions. However, those methods are only able to capture correlations. Thus, they may also focus on confounding information^71^. In order to prevent this from happening, the integration of a causal structure is essential, or via effective interpretability-guided inductive biases as reported recently in Mahapatra et al.^72,73^. For clinical applications where personal characteristics are exposed, primarily if these systems are used for educational purposes, where the images are shown to unauthorized personnel, it is extremely important to anonymize the retrieved cases before showing them. Even though Montenegro et al.^69,74^ already explored the use of privacy-preserving methods to anonymize medical images, further research is required in order to improve realism and preservation of clinical information. Finally, it is also relevant to explore federated learning settings for this particular purpose, as the training process would benefit considerably from using different datasets acquired with different scanners and representing different population characteristics.

## Data Availability

The data that support the findings of this study are available from PhysioNet (https://physionet.org/content/mimic-cxr-jpg/2.0.0/) but restrictions apply to the availability of these data, which were used under license for the current study, and so are not publicly available. Data and ranking annotations performed by the radiologists are however available from the authors upon reasonable request (through the following email contact: wilson.j.silva@inesctec.pt) and with permission of PhysioNet.
