# Author Correction: Computer-aided diagnosis through medical image retrieval in radiology

**DOI:** 10.1038/s41598-023-28523-1

**Published:** 2023-01-26

**Authors:** Wilson Silva, Tiago Gonçalves, Kirsi Härmä, Erich Schröder, Verena Carola Obmann, María Cecilia Barroso, Alexander Poellinger, Mauricio Reyes, Jaime S. Cardoso

**Affiliations:** 1 INESC TEC, Porto, Portugal; 2 Faculty of Engineering, University of Porto, Porto, Portugal; 3 Department of Diagnostic, Interventional and Pediatric Radiology, Inselspital, Bern University Hospital, University of Bern, Bern, Switzerland; 4 ARTORG Center for Biomedical Engineering Research, University of Bern, Bern, Switzerland

Correction to: *Scientific Reports* 10.1038/s41598-022-25027-2, published online 01 December 2022

The original version of this Article contained an error in the Acknowledgements section.

“This work was partially funded by the Project TAMI—Transparent Artificial Medical Intelligence (NORTE-01-0247-FEDER-045905) financed by ERDF—European Regional Fund through the North Portugal Regional Operational Program—NORTE 2020 and by the Portuguese Foundation for Science and Technology—FCT under the CMU—Portugal International Partnership, and also by the Portuguese Foundation for Science and Technology—FCT within PhD grants SFRH/BD/139468/2018 and 2020.06434.BD. The authors thank the Swiss National Science Foundation grant number 198388, as well as the Lindenhof foundation for their grant support.”

now reads:

“This work was supported by National Funds through the Portuguese Funding Agency, FCT–Foundation for Science and Technology Portugal, under Project LA/P/0063/2020, and also by the Portuguese Foundation for Science and Technology - FCT within PhD grants SFRH/BD/139468/2018 and 2020.06434.BD. The authors thank the Swiss National Science Foundation grant number 198388, as well as the Lindenhof foundation for their grant support.”

The original Article has been corrected.

